# Neuroplasticity of the left dorsolateral prefrontal cortex in patients with treatment-resistant depression as indexed with paired associative stimulation: a TMS–EEG study

**DOI:** 10.1093/cercor/bhad515

**Published:** 2024-01-09

**Authors:** Naotsugu Kaneko, Masataka Wada, Shinichiro Nakajima, Mayuko Takano, Keita Taniguchi, Shiori Honda, Masaru Mimura, Yoshihiro Noda

**Affiliations:** Department of Life Sciences, Graduate School of Arts and Sciences, The University of Tokyo, 3-8-1 Komaba, Meguro, Tokyo 153-8902, Japan; Department of Neuropsychiatry, Keio University School of Medicine, 35 Shinanomachi, Shinjuku, Tokyo 160-8582, Japan; Department of Neuropsychiatry, Keio University School of Medicine, 35 Shinanomachi, Shinjuku, Tokyo 160-8582, Japan; Department of Neuropsychiatry, Keio University School of Medicine, 35 Shinanomachi, Shinjuku, Tokyo 160-8582, Japan; Teijin Pharma Limited, 4-3-2 Asahigaoka, Hino, Tokyo 191-8512, Japan; Department of Neuropsychiatry, Keio University School of Medicine, 35 Shinanomachi, Shinjuku, Tokyo 160-8582, Japan; Department of Neuropsychiatry, Keio University School of Medicine, 35 Shinanomachi, Shinjuku, Tokyo 160-8582, Japan; Department of Neuropsychiatry, Keio University School of Medicine, 35 Shinanomachi, Shinjuku, Tokyo 160-8582, Japan; Department of Neuropsychiatry, Keio University School of Medicine, 35 Shinanomachi, Shinjuku, Tokyo 160-8582, Japan

**Keywords:** dorsolateral prefrontal cortex, neuroplasticity, paired associative stimulation, TMS–EEG, treatment-resistant depression

## Abstract

Major depressive disorder affects over 300 million people globally, with approximately 30% experiencing treatment-resistant depression (TRD). Given that impaired neuroplasticity underlies depression, the present study focused on neuroplasticity in the dorsolateral prefrontal cortex (DLPFC). Here, we aimed to investigate the differences in neuroplasticity between 60 individuals with TRD and 30 age- and sex-matched healthy controls (HCs). To induce neuroplasticity, participants underwent a paired associative stimulation (PAS) paradigm involving peripheral median nerve stimulation and transcranial magnetic stimulation (TMS) targeting the left DLPFC. Neuroplasticity was assessed by using measurements combining TMS with EEG before and after PAS. Both groups exhibited significant increases in the early component of TMS-evoked potentials (TEP) after PAS (*P* < 0.05, paired *t*-tests with the bootstrapping method). However, the HC group demonstrated a greater increase in TEPs than the TRD group (*P* = 0.045, paired *t*-tests). Additionally, event-related spectral perturbation analysis highlighted that the gamma power significantly increased after PAS in the HC group, whereas it was decreased in the TRD group (*P* < 0.05, paired *t*-tests with the bootstrapping method). This gamma power modulation revealed a significant group difference (*P* = 0.006, paired *t*-tests), indicating an inverse relationship for gamma power modulation. Our findings underscore the impaired neuroplasticity of the DLPFC in individuals with TRD.

## Introduction

Major depressive disorder (MDD) is among the most prevalent mental disorders, with an estimated 300 million individuals suffering from depression at the global level (World Health Organization 2017). Approximately, 30% of those with MDD experience treatment-resistant depression (TRD) (Rush et al. 2006; Gaynes et al. 2011). TRD refers to a form of MDD that does not respond adequately to appropriate courses of at least 2 antidepressant medications. Individuals with TRD often face diminished work productivity, restricted activity, lower quality of life, and increased utilization of medical resources compared to treatment responders with MDD and the general population (Jaffe et al. 2019). Given the urgent need for more effective treatments, it is essential to elucidate the pathophysiology of TRD.

The pathophysiology of TRD is complex and not fully understood. However, emerging evidence suggests that the potential neural basis of TRD may be characterized by alterations in specific brain circuits. Neuroimaging studies have consistently identified structural and functional abnormalities in several brain regions of individuals with TRD (McGrath et al. 2013; Dunlop et al. 2017; Akil et al. 2018). Key regions, such as the prefrontal cortex, anterior insula, amygdala, and hippocampus, seem to be differentially affected in TRD compared to non-TRD. These observed alternations in brain circuits could be indicative of impaired neuroplasticity in TRD.

Neuroplasticity, a fundamental nature of the brain, refers to its dynamic capability to adapt its structural and functional organizations in response to external and internal stimuli, experiences, and learning. This adaptability has led to the proposition of the neuroplasticity hypothesis as a potential underlying factor in the pathophysiology of psychiatric disorders (Spedding et al. 2003), especially depression (Castrén 2005, 2013; Price and Drevets 2012). This hypothesis indicates that impaired neuroplasticity in the brain circuits would act as a shared and fundamental mechanism underlying the pathophysiology of depression. Supporting the hypothesis in MDD, both basic and clinical researches have demonstrated that depression is associated with the impairment of structural and functional neuroplasticities (Cooke and Bliss 2006; Duman and Aghajanian 2012; Duman et al. 2016; Appelbaum et al. 2023). For example, previous research has reported a decreased volume in brain regions that control mood and cognition, such as the prefrontal cortex and hippocampus, and a decreased number of neural synapses in these regions in association with depression (Duman and Aghajanian 2012). Alongside the impairment of structural neuroplasticity, electrophysiological indices such as long-term potentiation (LTP) and LTP-like plasticity are reduced in depression, which suggests the impairment of functional neuroplasticity (Cooke and Bliss 2006). Furthermore, a meta-analysis of neuroimaging studies identified spatially convergent structural and functional abnormalities in MDD (Gray et al. 2020), potentially attributable to impaired neuroplasticity. These insights have encouraged further research based on the neuroplasticity hypothesis to elucidate the MDD pathophysiology, and adopting a similar approach is essential for understanding the TRD pathophysiology.

Paired associative stimulation (PAS) is one of the transcranial magnetic stimulation (TMS) paradigms designed to noninvasively induce functional neuroplasticity of the cerebral cortex in vivo (Jannati et al. 2023). Neuroplasticity can be assessed by comparing the corticospinal excitability or cortical activity before and after PAS. The PAS paradigm plastically changes synapses at the target site based on the Hebbian rule, which resembles the spike-timing dependent plasticity mechanism of LTP. PAS generally involves the repeated pairing of a single-pulse electrical stimulation to the peripheral nerve and a single pulse of TMS over the cortex. Originally, PAS was introduced as a method to induce neuroplastic changes in the primary motor cortex (M1) (Stefan 2000). A previous study using the M1–PAS paradigm was the first to show impaired neuroplasticity of M1 in individuals with MDD compared with healthy controls (HCs) (Player et al. 2013). Based on the findings of M1–PAS, Rajji and Noda et al. subsequently extended the targeted brain regions to the dorsolateral prefrontal cortex (DLPFC) (Rajji et al. 2013; Noda et al. 2018), which is involved in working memory and executive processing (Miller and Cohen 2001) as well as depression (Berlim et al. 2013; Kedzior et al. 2015). They confirmed that PAS effects were also observed in brain regions other than M1 using TMS combined with EEG, called TMS–EEG (Pascual-Leone et al. 2011; Ferreri and Rossini 2013; Rogasch et al. 2017). TMS–EEG allows us to record immediate brain responses to TMS, offering a way to evaluate the effects of various interventions on brain regions other than M1. This capability facilitated the extension of the M1–PAS paradigm to the DLPFC, termed as the DLPFC–PAS paradigm.

The neuroplastic effects induced by the DLPFC–PAS paradigm could emerge only when the interstimulus interval (ISI) between the peripheral median nerve stimulation and cortical stimulation is adjusted to interact (Rajji et al. 2013), suggesting that the physiological mechanism underpinning the neuroplasticity induced by the DLPFC–PAS paradigm aligns with the spike-timing-dependent plasticity observed in the M1–PAS paradigm (Stefan 2000; Stefan et al. 2002; Wolters et al. 2003). A previous study employing the DLPFC–PAS paradigm found that individuals with Alzheimer’s disease exhibited decreased neuroplasticity in the DLPFC when compared with HC, and the degree of the neuroplasticity was associated with cognitive functioning only in individuals with Alzheimer’s disease (Kumar et al. 2017). Therefore, the DLPFC–PAS paradigm applying the TMS–EEG method can assess functional neuroplasticity in the DLPFC (Rajji et al. 2013; Loheswaran et al. 2017; Noda et al. 2018). The application of this paradigm to TRD could provide valuable insights into its pathophysiological basis.

In TRD, the DLPFC is not only the hub of cognitive functions, including executive function and working memory in normal physiology, but abnormalities in networks originating from this region have also been identified as the pathological basis of depression (Wada et al. 2022). Here, neuroplasticity is the key function that regulates the basis of those neurophysiological and pathophysiological processes. Additionally, the neuroplasticity of the DLPFC underpins its significant role in emotional regulation, influencing mood and affects through its interactions with limbic structures. The therapeutic potential of targeting the neuroplasticity of the DLPFC is further highlighted by its targeted use in repetitive TMS (rTMS) treatments in this region, which have been shown to alleviate symptoms of TRD (Somani and Kar 2019). Consequently, it is essential to explore the neuroplasticity of the left DLPFC.

Therefore, the objective of the present study was to investigate the differences in functional neuroplasticity of the DLPFC between individuals with TRD and HCs. We utilized TMS–EEG (Pascual-Leone et al. 2011; Ferreri and Rossini 2013; Rogasch et al. 2017) to assess the neuroplasticity in the left DLPFC through the DLPFC–PAS paradigm. Considering that neuroplasticity deficits have been reported in MDD compared to HC (Noda et al. 2018) and that the severity of depressive symptoms in TRD is often higher than in MDD, we hypothesized that TRD would exhibit more severe impaired neuroplasticity. Given that the nature of TRD remains even more unclear than that of MDD, we also posited that investigating the neuroplasticity of the DLPFC in TRD could provide valuable insight into the pathophysiology of TRD and potential therapeutic strategies.

## Materials and methods

### Participants

The present study was conducted at Keio University from 2017 to 2022. All individuals participated in the present study after providing written informed consent according to the Declaration of Helsinki. The Ethics Committee of Keio University School of Medicine approved all experimental procedures (approval number: 20170152). Thirty participants in the HC group and 60 participants in the TRD group were included in the study (Table 1). The recruitment details for each group are as follows.

**Table 1:** Participant demographics, experimental parameters, and count difference.

Characteristic^a^	Patients with TRD	HCs	Statistics^b^	
	*n* = 60	*n* = 30		
Age, yr	45.4 ± 11.9	45.6 ± 13.2	*t*(88) = 0.096	*P* = 0.92
Female, %	40	40	—	
Education, yr	15.3 ± 1.9	14.7 ± 2.1	*t*(88) = 1.50	*P* = 0.14
MMSE score	29.1 ± 1.4	28.5 ± 3.3	*t*(88) = 1.38	*P* = 0.17
Age at onset of TRD, yr	36.0 ± 15.6	—	—	
Duration of illness, yr	10.9 ± 9.2	—	—	
MADRS score	28.2 ± 7.2	—	—	
TMS intensity (120%RMT), %MSO	75.8 ± 11.7	73.8 ± 11.4	*t*(88) = 0.761	*P* = 0.45
Nerve stimulation intensity (300%ST), mA	1.35 ± 0.47	1.42 ± 0.46	*t*(88) = 0.710	*P* = 0.48
SSEP N20 latency, ms	17.5 ± 1.7	18.1 ± 1.2	*t*(88) = 1.52	*P* = 0.13
Count difference	9.48 ± 16.29	2.83 ± 5.45	U = 617	*P* = 0.014

MADRS, Montgomery–Åsberg Depression Rating Scale; MMSE, Mini-Mental State Examination; HC; healthy control; MSO, maximum stimulator output; RMT, resting motor threshold; SSEP, somatosensory-evoked potentials; TMS, transcranial magnetic stimulation; TRD, treatment-resistant depression; ST, sensory threshold.

^a^Values are mean ± SD or %.

^b^ *P*-values are not corrected.

We recruited individuals aged 18–85 years with depression, who received routine clinical care at Keio University Hospital. Specifically, they were diagnosed with MDD according to the Diagnostic and Statistical Manual of Mental Disorders, Fifth Edition (American Psychiatric Association 2013) and met the following inclusion criteria for TRD: (i) a history of treatment failure, as indicated by a score of ≥3 on the Antidepressant Treatment History Form (Sackeim 2001), from at least 1 previous standard antidepressant excluding venlafaxine and (ii) a current severity defined as a score of ≥18 on the Montgomery Åsberg Depression Rating Scale (MADRS) (Williams and Kobak 2008). In addition, the exclusion criteria for this study were as follows: (i) substance use disorder within the past 6 months, (ii) contraindication to TMS and MRI, (iii) unstable physical or neurological disorders, (iv) history of seizures or epilepsy, or (v) cognitive dysfunction as assessed by Mini-Mental State Examination (MMSE). Since all individuals with TRD in the present study were scheduled to participate in another clinical trial (jRCT: 032180188) in which the type and dose of antidepressant medication had to be adjusted, antidepressants were unified to venlafaxine, and their doses were adjusted from 150 mg/day to 225 mg/day, and other antidepressants were tapered or discontinued, with the 4-week lead-in period including safety monitoring for adverse events. After the medication adjustment period and lead-in observation period (i.e. 2 months after participation in the study), depression severity was reevaluated, and only individuals still exhibiting moderate or higher depression severity at that time were enrolled in the TRD group. In summary, the TRD group in the present study comprised individuals with depression who were unresponsive to appropriate courses of at least 2 antidepressant medications, aligning with the TRD definition.

As part of the clinicodemographic assessments, clinical details, such as medical history and years of education, were obtained through interviews. Trained psychiatrists and clinical psychologists assessed the depression severity of each participant in the TRD group using the MADRS and Hamilton Depression Scale-21.

HCs met inclusion criteria if they were aged between 18 and 85 years old and had no history of psychiatric disorders, as confirmed by certified psychiatrists. The same exclusion criteria were applied to both TRD and HC groups. We have matched the age (within a 5-year range) and sex between the TRD and HC groups as much as possible. The sample size was determined based on a previous study comparing TMS–EEG indices between individuals with MDD and HC (Voineskos et al. 2019). In the present study, the ratio of the TRD group to the HC group was set to 2:1, in line with our previously published work (Wada et al. 2022). In view of the fact that most previous TMS–EEG studies were conducted with a sample size of about 20 participants, we anticipated that our present study, with 30 participants in the HC group and 60 participants in the TRD group, would adequately detect group differences in the TMS–EEG neurophysiological indices by the DLPFC–PAS paradigm.

### Study design

**Fig. 1:**
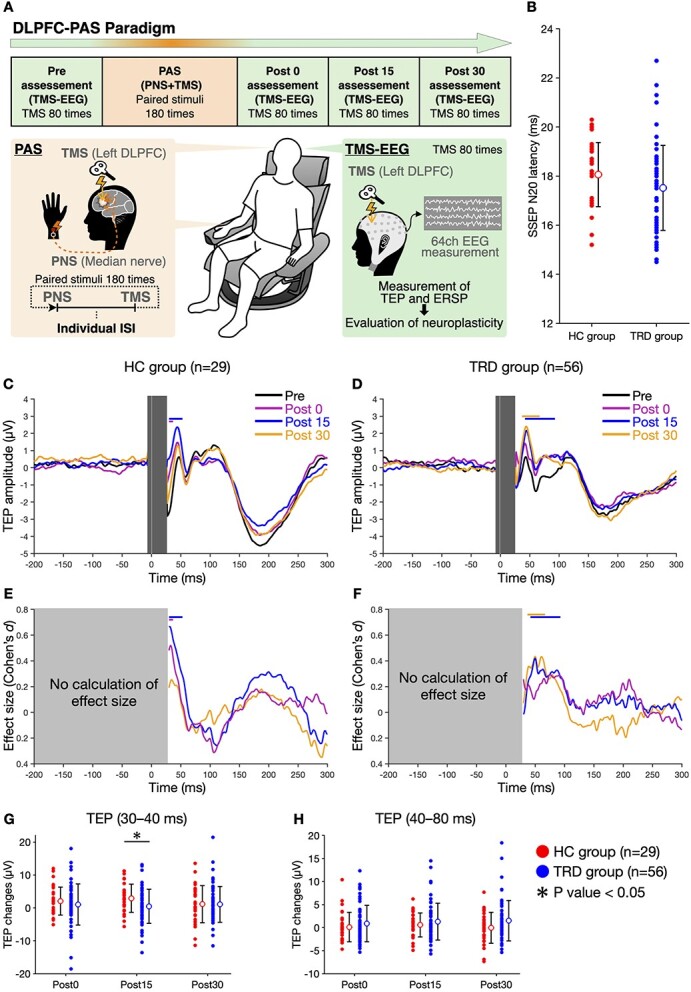
Panel A represents an overview of the DLPFC–PAS paradigm. To evaluate the neuroplasticity of the left DLPFC, participants underwent the DLPFC–PAS paradigm, involving repeated paired stimuli of PNS and TMS and TMS–EEG measurement. PAS consisted of 180 repeated paired stimuli of PNS to the median nerve and TMS over the left DLPFC. ISIs between PNS and TMS during the PAS were set based on the latency of SSEPs individually. We assessed the neuroplasticity using measurements combining TMS over the left DLPFC with EEG (i.e. TMS–EEG) before, 0, 15, and 30 min after the PAS. In TMS–EEG, TEP and ERSP were measured to evaluate the neuroplasticity. Panel B indicates a scatter plot of the latency of the SSEP N20 components. The filled plots display individual data in the HC and TRD groups. The unfilled circles and error bars represent the mean values and SDs, respectively. There was no significant difference in the SSEP latency between the groups (*P*-value < 0.05). Panels C–F indicate the average waveforms of TEP of the AF3, F1, F3, F5, and F7 channels corresponding to the stimulation site (i.e. the left DLPFC) with ESs. The left and right columns indicate the average TEP (B and C) with ESs (D and E) for the HC (*n* = 29) and TRD groups (*n* = 56), respectively. Black, purple, blue, and yellow lines represent the average waveforms of TEP with ESs at the Pre, Post0, Post15, and Post30 measurement points, respectively. The horizontal lines marked above the TEP waveforms correspond to the color of the TEP waveform and indicate the time when there were significant differences in the TEP (the FDR-corrected *P*-value < 0.05) between before (Pre) and after the DLPFC–PAS paradigm (Post0, Post15, and Post30). G and H indicate a scatter plot of TEP changes from 30 ms to 40 ms poststimulation and from 40 ms to 80 ms poststimulation, respectively. The red and blue plots display individual data in the HC and TRD groups, respectively. There was a significant group difference in TEP changes from 30 ms to 40 ms poststimulation at Post15; the TEP increase in the TRD group was significantly smaller than in the HC group (*P*-value < 0.05). PNS, peripheral nerve stimulation.

An overview of the DLPFC–PAS paradigm is shown in Fig. 1A. Every participant underwent the TMS–EEG measurement with the DLPFC–PAS paradigm. Before the experiment, MRI was scanned to identify the stimulus location for the TMS–EEG measurement with the DLPFC–PAS paradigm. Participants sat in a comfortable chair and were instructed to remain relaxed throughout the experiment. The DLPFC–PAS consisted of a single-pulse electrical stimulation to the peripheral median nerve located in the right wrist, immediately followed by a single-pulse TMS targeting the left DLPFC. ISIs between the median nerve stimulation and the TMS during the PAS were individually adjusted based on the latency of the somatosensory-evoked potentials (SSEPs). TMS–EEG measurements were conducted before, 0 min, 15 min, and 30 min after the DLPFC–PAS (Pre, Post0, Post15, and Post30). To evaluate the neuroplasticity of the DLPFC, we compared the data from the measurements between pre-PAS and post-PAS interventions. Subsequent sections delve deeper into these procedures.

### Preparation for identification of stimulus location

MRI data, individual high-resolution T1-weighted images, was used to identify the stimulus site. These images were obtained for each participant using a 3T Siemens Prisma scanner equipped with a 32-channel head coil and scanned T1-weighted magnetization-prepared rapid acquisition with gradient echo images. The acquisition parameters were as follows: echo time = 2.08 ms, repetition time = 1,620 ms, inversion time = 1,000 ms, flip angle = 8°, the field of view = 232 mm, matrixes = 186 × 192, and slice thickness = 1.25 mm.

### Electroencephalography

EEG data were recorded using a TMS-compatible 64-channel EEG amplifier equipped with a sample-and-hold circuit system (TruScan LTl; Deymed Diagnostic Ltd.). The data were sampled at a rate of 3 kHz. EEG recordings were made using an EEG cap fitted with silver cling electrodes. The arrangement of the 64 electrodes was based on the international 10–10 system. The ground electrode was placed on the left earlobe, while the reference electrode was positioned on the right earlobe. The impedance between the scalp and the electrodes was maintained <5 kΩ.

### Transcranial magnetic stimulation

We used the DuoMAG MP stimulator (DEYMED Diagnostic Ltd., Hronov, Czech Republic) that delivered a monophasic TMS through a figure-of-8 butterfly coil with 2 × 70 mm diameter windings (DuoMAG 70BF; DEYMED Diagnostic Ltd.). We imported an individual high-resolution T1-weighted image of each participant into a TMS neuronavigational system (Brainsight Rogue Research Inc., Montréal, Québec, Canada). Next, we manually identified the anterior and posterior commissures, interhemispheric plane, and brain bounds and performed registration to Montreal Neurological Institute (MNI) space. 3D reconstructions of the brain and scalp of each participant were obtained from MRI to define the virtual brain and scalp targets. 3D reconstructions of the brain and scalp of each participant were obtained from MRI to define the virtual brain and scalp targets. The optimal stimulus location for the left DLPFC was then identified by using MNI coordinates: *x* = −38, *y* = 44, and *z* = 26. The MNI coordinates used in the present study were representative of those used in TMS studies targeting the left DLPFC (Fox et al. 2012; Cardenas et al. 2022). In the experiment, coregistration was performed based on the digitized anatomical landmarks (the left and right tragus, nasion, and tip of the nose). The MRI-guided neuronavigation system enabled us to determine the target location (i.e. the left DLPFC) on the individual brain and to continuously monitor the coil’s relative position in real time. The TMS coil was positioned on the scalp at the identified location (MNI coordinates: *x* = −38, *y* = 44, and *z* = 26) with a 45° angle to the midline. To mitigate the influences of bone conduction of the clicking sound and coil vibration, a foam was placed directly beneath the coil during the experiment (Ter Braack et al. 2015).

EMG recordings were recorded from the muscle belly of the right first dorsal interosseous muscle with bipolar Ag/AgCl surface electrodes (Kendall H124SG, Covidien Medtronic) after cleaning the skin with alcohol. With the EEG cap in place, TMS was applied to the left M1 to determine the stimulus intensity of the TMS to the left DLPFC. Initially, we identified the optimal coil position, or “hotspot,” corresponding to the M1 site innervating the target right-hand muscle where the largest peak-to-peak amplitudes of motor-evoked potentials were elicited from the target muscle. Subsequently, the resting motor threshold was determined at the hotspot. This threshold was defined as the minimal TMS intensity required to produce the motor-evoked potential amplitudes of ≥50 μV in the relaxed target muscle in at least 50% of consecutive trials. For the DLPFC–PAS paradigm, as measured by TMS–EEG, the TMS intensity was set to 120% of the identified resting motor threshold (Noda et al. 2018).

### Median nerve stimulation

In this experiment, the median nerve stimulation at the right wrist was performed percutaneously by applying constant-current high-voltage pulses for a short time with the DS7A device (Digitimer model DS7A, Digitimer Ltd., United Kingdom) as part of the DLPFC–PAS paradigm. A standard bar electrode was located over the median nerve at the right wrist, with the cathode placed proximally. Stimulation pulses to the peripheral median nerve were monophasic, lasting 200 μs, and the stimulus intensity was set to 3 times the sensory threshold (Noda et al. 2018).

### Determination of the N20 latency in the SSEPs

The ISIs between the median nerve stimulation and TMS to the left DLPFC were determined based on the latency of the SSEP N20 components, accounting for the interindividual differences in the conduction time of the sensory afferent input to the cortex (Noda et al. 2016, 2018; Cash et al. 2017). Before the TMS–EEG premeasurement, we recorded EEG data when the median nerve was stimulated 200 times at a frequency of 2 Hz. The EEG data were analyzed using TruScan Acquisition (TruScan LTl; DEYMED Diagnostic s.r.o., Czech Republic). In this analysis, EEG signals for each channel were epoched between 105 ms prestimulus and 400 ms poststimulus, filtered with a high-pass filter set at 1 Hz, baseline-corrected based on the segment from −105 ms to −5 ms prestimulus, and then averaged. Subsequently, we visually identified the individual N20 latency of SSEPs at the C3, CP1, and CP5 electrodes. The sensory input signals of peripheral nerve stimulation are relayed to the thalamus via the spinothalamic tract and projected to the somatosensory cortex. A portion of the sensory signals reaching the somatosensory cortex propagates indirectly to the DLPFC via (i) the cortico-cortical propagation, while the other signals project directly to the DLPFC from the middle of the spinothalamic tract via (ii) the thalamocortical circuit (Miller and Cohen 2001). The individual ISI for the DLPFC–PAS paradigm was set to be 4 ms longer than the determined N20 latency (i.e. individual N20 latency +4 ms). This setting ensures the most pronounced short-latency afferent inhibition in the left DLPFC (Noda et al. 2016), in line with the mechanism of the DLPFC–PAS paradigm.

### Combined TMS–EEG measurement

The TMS–EEG measurement was conducted before 0 min, 15 min, and 30 min after the DLPFC–PAS paradigm (labeled as Pre, Post0, Post15, and Post30, respectively). During each measurement, EEG was recorded when delivering TMS to the left DLPFC. In a single measurement, participants received 80 single-pulse TMS with a jittering of 500 ms, resulting in intertrial intervals ranging from 4.5 s to 5.5 s. This interval was sufficient to recharge the TMS coil. They were instructed to listen to white noise via an ear plug sound stimulation system throughout the session (i.e. sound masking), which minimized auditory-evoked potentials elicited by the clicking sound of TMS (Ter Braack et al. 2015). Before initiating the TMS–EEG measurement, the white noise volume was individually adjusted to a level where the TMS click sound was effectively masked.

### Paired associative stimulation

The present study applied the DLPFC–PAS paradigm to induce neuroplasticity in the left DLPFC. Within this paradigm, participants underwent 180 paired stimuli, each comprising median nerve stimulation followed by TMS to the left DLPFC with ISIs tailored to each individual (the specifics of each stimulus parameter are detailed above). The intervals of paired stimuli ranged from 4.5 s to 5.5 s, which was sufficient for TMS coil recharge. The participants were instructed to mentally count the number of median nerve stimulations and answer the number when asked by an experimenter during the DLPFC–PAS to keep their attention (Noda et al. 2018), which is assumed to robustly induce neuroplastic changes. This counting task was incorporated because counting the number of stimuli given to the right wrist is known to enhance neuroplastic changes, whereas delivering attention away from the stimulus attenuates these effects (Stefan et al. 2004). The absolute value of the discrepancy between the actual count and the participant’s count was termed as the count difference and served as an attention indicator during the DLPFC–PAS paradigm (Kumar et al. 2017).

### E‌EG analysis

The EEG data were analyzed offline using custom programs in MATLAB 2022a (MathWorks, Natick, MA, United States) that incorporate functions of the EEGLAB 2021.1 (Delorme and Makeig 2004) and TMS–EEG Signal Analyzer (TESA) v1.1.1 (Rogasch et al. 2017). One participant in the HC group was excluded due to the absence of post-PAS data.

####  

##### E‌EG preprocessing

Initially, the EEG data were epoched between −2,000 ms before (pre-TMS) and 2,000 ms after TMS (post-TMS) and were baseline-corrected by subtracting the average signal amplitude from −500 ms to −150 ms pre-TMS. Then, channel electrodes and epochs with abnormal amplitudes and noises, resulting from nonbrain activity factors, were automatically identified and removed. Zero-padding was applied to the EEG data from −5 ms to 30 ms pre- and post-TMS to eliminate the TMS pulse artifacts. The EEG data were then downsampled from 3 kHz to 1 kHz.

Two rounds of independent component analysis (ICA) were conducted. The first round of fast ICA on the preprocessed EEG data aimed to remove the physical decay artifact components of TMS on the EEG. The EEG data were then band-pass filtered between 0.5 Hz and 100 Hz using a forward-backward fourth-order Butterworth filter and were notch-filtered between 48 Hz and 52 Hz. The second round of ICA, utilizing the “runica” function in EEGLAB (Makeig et al. 1996), differentiated the EEG data into brain activity and nonbrain-related artifacts. The “tesa_compselect” function in the TESA toolbox was employed to discard the independent components associated with the noisy electrodes, blinks, eye movements, and muscle artifacts. For the remaining components of each participant, we estimated equivalent current dipoles within a standardized 3-shell boundary element head model based on the MNI standard brain using the DIPFIT toolbox in EEGLAB and an electrode position template based on the MNI head model (Oostenveld and Oostendorp 2002; Delorme et al. 2012). Subsequent analysis only considered independent components whose best-fit equivalent current dipoles were located within the head, accounting for >85% of the variance found in the scalp (Shirazi and Huang 2019; Kaneko et al. 2021). Independent components not meeting this threshold were identified as eye or muscle artifact components and were removed (Jung et al. 2000). The further analysis included participants with ≥6 components remaining from the analysis so far (ranging between 6 and 22 components in the HC group and between 6 and 21 components in the TRD group). Consequently, TMS–EEG data from 29 participants (97%) in the HC group and 56 (93%) in the TRD group were retained for further analyses. Lastly, we rereferenced the data to the average (overall electrodes) for sensor-based EEG analysis.

##### Sensor-based EEG analysis for the left DLPFC

The present study focused on neuroplasticity in the left DLPFC. Initially, we verified the PAS effects on TMS-evoked potentials (TEP) in the left DLPFC, calculated from the mean TEP amplitudes of the AF3, F1, F3, F5, and F7 electrode sites. Subsequently, we computed the event-related spectral perturbation (ERSP), representing the frequency power modulation induced by single-pulse TMS to the left DLPFC, using EEGLAB functions (Delorme and Makeig 2004). For time-frequency analysis, a 2-cycle standard Morlet wavelet transform was applied at each frequency from 4 Hz to 48 Hz, and ERSP was baseline-normalized with power from −500 ms to −100 ms pre-TMS. The baseline-normalized ERSPs of the AF3, F1, F3, F5, and F7 electrode channels were then averaged.

### Statistical analyses

#### Statistical analysis for participant demographics and experimental parameters

The Student’s *t*-test was performed to compare demographics (age, years of education, and MMSE scores) and experimental parameters (TMS and nerve stimulation intensities and the latency of the SSEP N20) between the HC and TRD groups. The Mann–Whitney U test was employed to investigate the differences in counting errors during the DLPFC–PAS paradigm between the 2 groups.

#### Statistical analysis for PAS effects in each group

To assess the effects of the DLPFC–PAS paradigm on TEP and ERSP within the HC and TRD groups, the following steps were taken. First, TEP in the time range of 30 ms and 300 ms after TMS (271 time points) was compared between before (Pre) and after the DLPFC–PAS (Post0, Post15, and Post30) using paired *t*-tests and a 2,000-iteration bootstrapping method. For the baseline-normalized ERSP, the significant region of different frequency power after TMS (30–300 ms) from the baseline frequency power before TMS (−500 ms to −100 ms) was computed for each measurement (Pre, Post0, Post15, and Post30) using the 2,000-iteration bootstrap method. Differences in baseline-normalized ERSPs between before (Pre) and after (Post0, Post15, and Post30) the DLPFC–PAS were determined using paired *t*-tests and the 2,000-iteration bootstrap method. *P*-values were corrected using the false discovery rate (FDR) method, and Cohen’s *d* value was calculated as the effect size (ES) for both TEP and ERSP. For all statistical tests, the significance level was set at *P* < 0.05. Thresholds for interpreting Cohen’s *d* values were set to 0.2, 0.5, and 0.8 for small, medium, and large ESs, respectively.

#### Statistical analysis of group differences in the PAS effect

The changes in TEP and ERSP after the DLPFC–PAS paradigm were compared between the HC and TRD groups. Time-frequency interest for these comparisons was determined based on the statistical results of the PAS effects within each group; that is, the between-group comparisons focused on the time and frequency where significant PAS effects were observed. An analysis of covariance (ANCOVA) was performed with changes in TEP and ERSP over the time-frequency interest as dependent variables, the group (HC vs. TRD) as the independent fixed factor, age of participants, and count difference as the covariates to account for potential attentional confounding during the DLPFC–PAS paradigm. For all statistical tests, the significance level was set at *P* < 0.05. Data are described as mean ± SD.

Pearson's correlation analysis was performed to examine the relationship between pre-TMS TME-EEG metrics (i.e. TEP and ERSP over the time-frequency interest) before the DLPFC–PAS paradigm and the modulatory changes of these metrics by the PAS. For a comparison of correlation coefficients between the HC and TRD groups, Fisher’s *Z* transformation was used to calculate the *z*-score from the correlation coefficients and *P*-values corresponding to the *z*-score.

The effect of PAS on TEP and ERSP within each group was statistically analyzed using MATLAB 2022a (MathWorks), equipped with the bootstrap method. Comparisons of demographics (using Student’s *t*-test and Mann–Whitney U test) and PAS effects (using ANCOVA and Pearson’s correlation analysis) between groups were conducted with the open-source software jamovi v. 2.3.26.0 (https://www.jamovi.org).

## Results

### Participant demographics and experimental parameters

Table 1 outlines the demographics of participants in the TRD and HC groups. Age and sex were matched between the 2 groups. Education years and MMSE scores showed no significant differences between the groups. Table 1 also provides details on the experimental parameters and count differences in the DLPFC–PAS paradigm. There were no significant differences in the intensities of TMS and nerve stimulation (both *P* > 0.05, Table 1) and the latency of the SSEP N20 between the 2 groups (*P* > 0.05, Fig. 1B). However, the TRD group had a higher count difference than the HC group.

### Changes in TEP after the DLPFC–PAS paradigm and their differences between groups

Figure 1B–E displays the average TEP calculated from the electrodes corresponding to the stimulation site (i.e. the left DLPFC) with the ES index for each measurement point in both groups. In the HC group, the TEP between 30 ms and 40 ms poststimulation was significantly increased at Post0 and Post15 compared to Pre (Fig. 1C). In the TRD group, the TEP between 40 ms and 80 ms poststimulation was significantly increased at Post15 and Post30 compared to Pre (Fig. 1D). The ES, as indexed by Cohen’s *d*, revealed a moderate TEP increase in the HC group (Fig. 1E) and a small one in the TRD group (Fig. 1F). These results indicated that the DLPFC–PAS enhanced the early TEP components in the left DLPFC for both HC and TRD groups.

Comparisons between groups focused on TEP changes from 30 ms to 40 ms poststimulation and from 40 ms to 80 ms poststimulation, where significant TEP increases were observed in each group. The ANCOVA revealed a significant difference between the HC and TRD groups in the TEP increase from 30 ms to 40 ms poststimulation at Post15 (*F*_1, 81_ = 4.00, *P* = 0.049), but not at Post0 (*F*_1, 81_ = 0.695, *P* = 0.407), or Post30 (*F*_1, 81_ = 0.0145, *P* = 0.904), after controlling for the age of participants and count difference as covariates. Post hoc *t*-tests indicated that the TEP increase in the TRD group was significantly smaller than that in the HC group (Fig. 1G: *t*_81_ = 2.00, *P* = 0.049, *d* = 0.471). Furthermore, the ANCOVA showed no significant differences between the groups for the TEP change from 40 ms to 80 ms poststimulation at Post0 (*F*_1, 81_ = 1.27, *P* = 0.262), Post15 (*F*_1, 81_ = 0.938, *P* = 0.336), or Post30 (*F*_1, 81_ = 2.92, *P* = 0.091) (Fig. 1H).

### Changes in ERSP after the DLPFC–PAS paradigm and their differences between groups

**Fig. 2:**
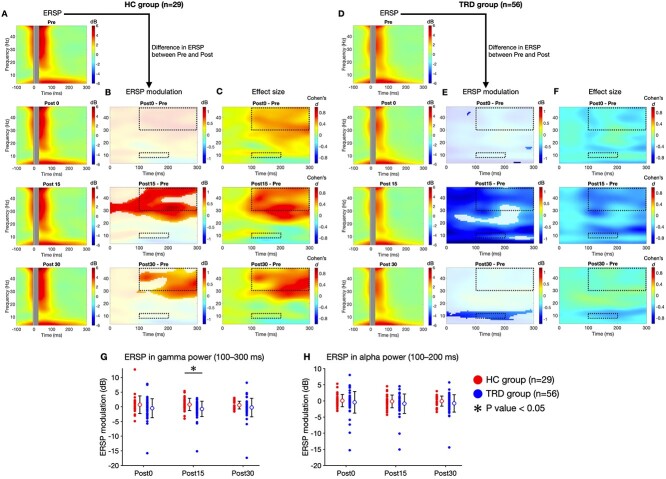
A and D indicate the average ERSPs of the AF3, F1, F3, F5, and F7 channels corresponding to the stimulation site (i.e. the left DLPFC) before (Pre) and after the DLPFC–PAS paradigm (Post0, Post15, and Post30) for the HC group (*n* = 29) and the TRD group, respectively. The average ERSPs normalized to the baseline frequency power before TMS (from −500 to −100 ms) are presented. The ERSPs at the Pre, Post0, Post15, and Post30 measurement points are shown from top to bottom. B and E indicate ERSP modulation calculated by the average ERSPs after the DLPFC–PAS paradigm (Post0, Post15, and Post30) minus those before the paradigm (Pre) from top to bottom. Color scales for A, B, D, and E represent the value of decibel power (dB). Nonsignificant values are masked in white (the FDR-corrected *P*-value > 0.05) in ERSP modulation. C and F show the ESs. Color scales for C and F represent Cohen’s *d* values. The areas enclosed by the dashed line were used for between-group comparisons. G and H indicate a scatter plot of ERSP modulation within the gamma (30–48 Hz) and alpha bands (8–12 Hz) after 100 ms poststimulation, respectively. The filled plots display individual data in the HC and TRD groups. The unfilled circles and error bars represent the mean values and SDs, respectively. There was a significant group difference in ERSP modulation within the gamma from 100 ms to 300 ms poststimulation at Post15; the ERSP gamma modulation was significantly more pronounced in the HC group compared to the TRD group (*P*-value < 0.05).

Figure 2A and D presents the average ERSP plots from the electrodes corresponding to the stimulation site (i.e. the left DLPFC) for each measurement point in both groups. Figure 2B and E depicts the differences in the average ERSP before and after the DLPFC–PAS paradigm (Post0, Post15, and Post30 minus Pre) for the HC and TRD groups, respectively. Figure 2B highlights an increase in gamma band power at Post15 and Post30 for the HC group. The increase became evident after 100 ms poststimulation, exhibiting medium to large ESs (Fig. 2C). Conversely, Fig. 2E shows a significant decrease in frequency power in all analyzed frequency bands (i.e. 4–48 Hz) at Post15 for the TRD group. Additionally, a significant decrease in the alpha band power was also detected at Post30. These power decreases were especially prominent in the gamma band between 100 ms and 150 ms poststimulation at Post15 and in the alpha band after 100 ms poststimulation at both Post15 and Post30, exhibiting moderate ESs (Fig. 2F). These results highlighted contrasting modulations after the DLPFC–PAS paradigm: The HC group exhibited an increase in frequency power, whereas the TRD group showed a decrease, predominantly in the gamma band.

Comparisons between the groups focused on changes in the ERSP within the gamma (30–48 Hz) and alpha bands (8–12 Hz) after 100 ms poststimulation, where each group exhibited significant ERSP changes (as outlined by the dash lines in Fig. 2B and E). The ANCOVA revealed a significant difference between the HC and TRD groups in ERSP gamma modulation from 100 ms to 300 ms poststimulation at Post15 (*F*_1, 81_ = 7.67, *P* = 0.007), controlling for the age of participants and count difference as covariates. Post hoc *t*-tests indicated that the ERSP gamma modulation was significantly more pronounced in the HC group compared to the TRD group (Fig. 2G: *t*_81_ = 2.77, *P* = 0.007, *d* = 0.653). By contrast, there was no significant group difference in the ERSP gamma modulation from 100 ms to 300 ms poststimulation at Post0 (*F*_1, 81_ = 2.50, *P* = 0.117) or Post30 (*F*_1, 81_ = 1.98, *P* = 0.163). Furthermore, the ANCOVA revealed no significant group differences in the ERSP alpha modulation from 100 ms to 200 ms poststimulation at Post0 (*F*_1, 81_ = 0.146, *P* = 0.703), Post15 (*F*_1, 81_ = 1.36, *P* = 0.248), or Post30 (*F*_1, 81_ = 1.46, *P* = 0.231) (Fig. 2H).

There were significant negative correlations between the pre-TMS values and modulatory changes of these metrics by the PAS in all TEP and ERSP metrics for each group (Table 2). For TEPs, higher pre-TMS values before the PAS resulted in a lower increase, while for ERSPs, higher pre-TMS power before the PAS resulted in greater power inhibition. For TEP between 40 ms and 80 ms at Post15 and ERSP in the gamma band at Post30, there were significant differences in the correlation coefficients between the groups (Table 2).

**Table 2:** Pearson’s correlation coefficients (*r*) and *P*-values (*P*) between pre-PAS TEP and ERSP metrics and modulation of TEP and ERSP and group differences in correlation coefficients.

Pre-PAS		HC group	TRD group	Group difference
metrics		(*n* = 29, df = 27)	(*n* = 56, df = 54)	
TEP	Pre–Post0	*r* = −0.702	*r* = −0.570	*z*-score = 0.934
30–40 ms		*P* <0.001	*P* <0.001	*P* = 0.350
	Pre–Post15	*r* = −0.585	*r* = −0.679	*z*-score = 0.657
		*P* <0.001	*P* < 0.001	*P* = 0.511
	Pre–Post30	*r* = −0.566	*r* = −0.576	*z*-score = 0.062
		*P* < 0.001	*P* < 0.001	*P* = 0.951
TEP	Pre–Post0	*r* = −0.730	*r* = −0.631	*z*-score = 0.775
40–80 ms		*P* < 0.001	*P* < 0.001	*P* = 0.438
	Pre–Post15	*r* = −0.411	*r* = −0.727	*z*-score = 2.028
		*P* = 0.027	*P* < 0.001	*P* = 0.043
	Pre–Post30	*r* = −0.499	*r* = −0.687	*z*-score = 1.229
		*P* = 0.006	*P* < 0.001	*P* = 0.219
ERSP in	Pre–Post0	*r* = −0.465	*r* = −0.621	*z*-score = 0.931
gamma power		*P* = 0.011	*P* < 0.001	*P* = 0.352
	Pre–Post15	*r* = −0.446	*r* = −0.574	*z*-score = 0.726
		*P* = 0.015	*P* < 0.001	*P* = 0.468
	Pre–Post30	*r* = −0.799	*r* = −0.962	*z*-score = 3.660
		*P* < 0.001	*P* < 0.001	*P* < 0.001
ERSP in	Pre–Post0	*r* = −0.572	*r* = −0.419	*z*-score = 0.852
alpha power		*P* = 0.001	*P* = 0.001	*P* = 0.394
	Pre–Post15	*r* = −0.715	*r* = −0.426	*z*-score = 1.847
		*P* < 0.001	*P* = 0.001	*P* = 0.065
	Pre–Post30	*r* = −0.527	*r* = −0.759	*z*-score = 1.704
		*P* = 0.003	*P* < 0.001	*P* = 0.089

ERSP, event-related spectral perturbation; HC. healthy control; PAS, paired associative stimulation; TEP, TME-evoked potential; TMS, transcranial magnetic stimulation; TRD, treatment-resistant depression.

## Discussion

In the present study, we employed the DLPFC–PAS paradigm to investigate the differences in functional neuroplasticity of the left DLPFC between the TRD and HC groups. Both groups showed plastic changes in the cortical response of the left DLPFC to TMS after the paradigm; however, the latency and extent of the changes were different. In the TRD group, compared to the HC group, the TEP latency of the facilitation was later, and the TEP increase in the early component was smaller (Fig. 1C–H). Furthermore, ERSP in the TRD group exhibited power suppression (Fig. 2E), contrasting with the power facilitation observed in the HC group (Fig. 2B). Notably, a significant inverse power modulation between the TRD and HC groups was identified in the gamma band. Our findings showed that reduced neuroplasticity in the DLPFC of TRD was also manifested as reduced gamma oscillations, which is in alignment with our hypothesis.

### Increases in TEP after the DLPFC–PAS paradigm and their group differences

We assessed the neuroplasticity of the DLPFC by using the DLPFC–PAS paradigm and TMS–EEG measurement established in previous studies (Rajji et al. 2013; Noda et al. 2018). Based on these studies, this paradigm is known to induce facilitatory effects on the DLPFC, similar to the LTP effect induced by spike-timing dependent plasticity. The present results showed that the paradigm increased the early component of TEP in the HC group (Fig. 1C), confirming the successful induction of LTP-like change induced by spike-timing-dependent plasticity in the DLPFC. Similarly, the TRD group exhibited an increase in the early component of TEP (Fig. 1D), validating the DLPFC–PAS paradigm used in the present study for comparing the neuroplasticity across the groups.

For the latency, the HC group exhibited increases in the early component of TEP between 30 ms and 40 ms poststimulation (Fig. 1C), whereas the TRD group showed increases between 40 ms and 80 ms poststimulation (Fig. 1D). Furthermore, our results showed group differences in the degree of modulation (Fig. 1G). The HC group exhibited larger ESs for TEP modulation in the early components at Post15 compared to the TRD group (Fig. 1E and F). Therefore, similar to the observed neuroplasticity deficits of the DLPFC in MDD (Noda et al. 2018), individuals with TRD also exhibited impaired neuroplasticity when compared to HCs. The TEP latency, modulated by the DLPFC–PAS paradigm, offers insights into neurotransmitter and receptor dysfunction. Several previous studies have proposed that each component of TEP is associated with various neurotransmitter receptor-mediated neurophysiological functions, depending on its latency (Ferreri et al. 2011; Rossini et al. 2015; Cash et al. 2017). Through pharmacological and electrophysiological profiling researches, mainly focusing on M1, the neurophysiology of TEP has been elucidated. For TEPs elicited by TMS to M1, typically positive peak P30 and P60 components are observed at latencies of about 30 ms and 60 ms poststimulus, and a negative peak N45 component is observed at a latency of around 45 ms poststimulus (Ilmoniemi and Kičić 2010; Ferreri et al. 2011). When linking the inhibition and facilitation of these TEP components to various neurotransmitter receptor functions, the N45 component is reported to reflect the neurophysiological functions mediated by the GABA_A_ receptor function (Premoli et al. 2014) and glutamate N-methyl-D-aspartate receptors (Belardinelli et al. 2021), while the P60 component is reported to be associated with the glutamate α-amino-3-hydroxy-5-methyl-4-isoxazolepropionic acid receptor-mediated neurophysiological functions (Belardinelli et al. 2021).

Similar to the M1, the early components of TEP, especially the P60 amplitude, reflect the involvement of GABA_A_ receptor-mediated inhibition or glutamatergic activity with short-interval intracortical inhibition and intracortical facilitation in the DLPFC (Cash et al. 2017). Furthermore, MDD is linked to domain-specific modulation of GABA_A_ receptor subunit composition and GABA concentration (Pehrson and Sanchez 2015) and to impaired neuroplasticity of the DLPFC (Noda et al. 2018). The balance between excitatory (mainly glutamatergic) and inhibitory (primarily GABAergic) neurotransmission is pivotal for various neuroplasticity forms. Therefore, the observed TEP modulation in the present study, with its different latencies and extent, may indicate GABAergic and glutamatergic dysfunctions, leading to impaired neuroplasticity of the DLPFC in individuals with TRD. Moreover, this interpretation aligns with magnetic resonance spectroscopy studies that demonstrated individuals with TRD had decreased levels of GABA compared to HC as well as individuals with non-TRD (Price et al. 2009). Moreover, rTMS responders with TRD exhibited increased levels of GABA following rTMS treatment to the DLPFC (Levitt et al. 2019).

### Opposite ERSP modulation between groups after the DLPFC–PAS paradigm in the gamma band

The ERSP results revealed distinct power modulation between the groups, suggesting impaired neuroplasticity in the DLPFC, as did the TEP results. The DLPFC–PAS paradigm increased gamma power in the HC group (Fig. 2B), while it decreased the overall frequency power in the TRD group (Fig. 2E). Interestingly, these findings highlighted contrasting ERSP modulation between the groups. Moreover, significant differences in the opposite ERSP modulation were observed in the gamma band from 100 ms to 300 ms poststimulation at Post15 between the groups (Fig. 2G). A previous study with an experimental design similar to the present study found significant differences in the modulation of delta, theta, and gamma frequency powers between the MDD and HC groups (Noda et al. 2018). The MDD group showed less potentiation of the delta, theta, and gamma frequency powers after the DLPFC–PAS paradigm compared to the HC group (Noda et al. 2018). It should be noted that this previous study recruited participants with MDD, including a portion of participants with TRD, and did not report a decrease in frequency power in MDD after the PAS paradigm. By contrast, the present study focused solely on participants with TRD, representing a more severe and treatment-resistant type of MDD. Considering ES, the difference in gamma power modulation between the TRD and HC groups in the present study (*d* = 0.65) was larger than that between the MDD and HC groups in the previous study (*d* = 0.42). Consequently, we found robust features in TRD that were clearly different from HC in terms of power reduction after the DLPFC–PAS paradigm. Our results, in light of previous DLPFC–PAS findings for MDD (Noda et al. 2018), suggest that the degree of neuroplasticity by the DLPFC–PAS paradigm decreases in a gradient in the order of HC, MDD, and TRD and that the neuroplasticity in the DLPFC may represent part of the pathophysiology, including treatment resistance, in depression. Furthermore, the DLPFC–PAS paradigm led to an increase in gamma frequency power in HC, which was not observed as a significant increase in MDD, but rather as a decrease in gamma frequency power in TRD, suggesting that the neurophysiological mechanisms involved in neuroplasticity may have been drastically altered in the TRD group.

The apparent opposite power modulation was identified in the gamma band, where there were significant differences in ERSP modulation between the groups.

Gamma oscillations are considered to be important in exploring the differences in cortical activity between individuals with MDD and HCs (Akar et al. 2015). For example, individuals with MDD exhibited decreased gamma oscillations in the frontal and parietal areas associated with emotional responses compared to HCs (Lee et al. 2010). Beyond localized effects, a recent study using the 40 Hz auditory steady-state response reported that impairments in the generation and maintenance of gamma oscillations reflect abnormalities in the distributed network across parietal to frontal regions in MDD (Chen et al. 2023). Therefore, the inverse power modulation in the gamma band in TRD, compared to HC, suggests that the impaired neuroplasticity of the DLPFC and the network containing it reflects the pathophysiology of TRD.

### Delayed effects of the DLPFC–PAS on TEP and ERSP

The present study performed TMS–EEG measurements at 3 time points after the DLPFC–PAS paradigm (i.e. Post0, Post15, and Post30). This approach was adopted because the PAS effects can vary due to the individual differences and pathological conditions and may not be necessarily pronounced immediately postintervention. Several previous studies conducted postassessments at these 3 time points to capture the time course of neuroplasticity and to avoid missing the PAS effects (Frantseva et al. 2008; Rajji et al. 2013; Noda et al. 2018). Our results showed that both groups had marked changes in TEP and ERSP 15 min after the DLPFC–PAS paradigm (Post15). Given the delayed effects of the PAS, experimental designs probing neuroplasticity, such as those employing PAS and rTMS, should carefully determine the number and timing of subsequent assessments.

### Relationship between the pre-TMS values and modulatory changes in TEP and ERSP metrics

We found significant negative correlations between the pre-TMS values and modulatory changes in all TEP and ERSP metrics by the PAS for each group (Table 2). These results suggest that pre-TMS values could predict the degree of modulation of TEP and ERSP. The negative correlation found in both the groups indicates that the relationship between the pre-TMS values and modulatory changes may not be specific to the TRD or HC groups but may be a feature of the EEG. For TEP between 40 ms and 80 ms at Post15 and ERSP in the gamma band at Post30, there were significant differences in the correlation coefficients between the groups (Table 2). However, modulation of these TEP and ERSP metrics did not show any significant group differences. Furthermore, there were no significant group differences in correlation coefficients for other parameters and time points (Table 2). Therefore, although a significant correlation was observed between the pre-TMS metrics and the degree of modulation of TEP and ERSP, pre-TMS metrics had little impact on the results of group differences in TEP and ERSP modulation.

### Limitations of the present study

Although the present study is the first to examine functional neuroplasticity in the left DLPFC of TRD by using the DLPFC–PAS paradigm and TMS–EEG measurement, there are several limitations. First, we only recruited individuals with TRD who had depressive symptoms, making it ambiguous whether our findings indicated state or trait markers of TRD. Second, in the present study, since individuals with TRD received the same type and dose of antidepressant, the effects of the antidepressant were rigorously controlled within the TRD group. However, in the comparison between the TRD and HC groups, we cannot rule out the possibility that the use of that antidepressant in the TRD group had a certain effect on the neural activity, which may have led to the differences in TEP and ERSP between the 2 groups. Third, the present study did not compare evoked cortical activity between active TMS with sham stimulation, and the evoked activity might not originate solely from the neuronal firing of the left DLPFC (stimulation site) since TMS elicits neural inputs from cutaneous sensation. Furthermore, since we set only the left DLPFC as the stimulation site, the present study may have only part of the pathophysiological basis of TRD. Future research using sham stimulation and TMS on other brain regions, such as the right DLPFC, will provide a more comprehensive picture of the pathophysiology of TRD. Lastly, we could not examine the relationship between the modulation of TEP and ERSP after the DLPFC–PAS paradigm and changes in clinical symptoms because the DLPFC–PAS was examined for the neurophysiological examination purposes at baseline and the clinical symptoms were not measured after the PAS paradigm. Clarification of such relationships will require longitudinal studies of some intervention to evaluate the neuroplasticity in the DLPFC in individuals with TRD.

## Conclusion

Our study highlighted the impaired neuroplasticity of the DLPFC in individuals with TRD, aligning with the neuroplasticity hypothesis for depression (Price and Drevets 2012; Castrén 2013). The observed impaired neuroplasticity in TRD showed the cortical gamma modulation opposite to that of HC in the DLPFC–PAS paradigm. These findings provide evidence for impaired neuroplasticity in the DLPFC of individuals with TRD and its potential role in cognitive dysfunction, contributing to the elucidation of the pathophysiology of TRD. Moreover, the application of the DLPFC–PAS paradigm to TRD may be a useful biomarker for the early diagnosis and treatment monitoring of TRD. Further research is needed to investigate how the impaired neuroplasticity changes with therapeutic treatments, such as rTMS treatment, along with the recovery of clinical symptoms and cognitive function, which may improve the personalized therapeutic approaches for this challenging disorder.

## Data Availability

TMS–EEG data and the MATLAB-based scripts used in the analyses are available upon reasonable request to the corresponding author (YN).

## References

[ref1] Akar SA, Kara S, Agambayev S, Bilgic V. Nonlinear analysis of EEG in major depression with fractal dimensions. 37th Annual International Conference of the IEEE Engineering in Medicine and Biology Society (EMBC). Presented in 2015 25–29 Aug:2015:7410–7413. 10.1109/EMBC.2015.7320104 26738004

[ref2] Akil H, Gordon J, Hen R, Javitch J, Mayberg H, McEwen B, Meaney MJ, Nestler EJ. Treatment resistant depression: a multi-scale, systems biology approach. Neurosci Biobehav Rev. 2018:84:272–288. 28859997 10.1016/j.neubiorev.2017.08.019 PMC5729118

[ref3] American Psychiatric Association . Diagnostic and statistical manual of mental disorders. 5th ed. Arlington (VA): American Psychiatric Association; 2013.

[ref4] Appelbaum LG, Shenasa MA, Stolz L, Daskalakis Z. Synaptic plasticity and mental health: methods, challenges and opportunities. Neuropsychopharmacology. 2023:48(1):113–120. 35810199 10.1038/s41386-022-01370-w PMC9700665

[ref5] Belardinelli P, König F, Liang C, Premoli I, Desideri D, Müller-Dahlhaus F, Gordon PC, Zipser C, Zrenner C, Ziemann U. TMS-EEG signatures of glutamatergic neurotransmission in human cortex. Sci Rep. 2021:11(1):8159. 33854132 10.1038/s41598-021-87533-z PMC8047018

[ref6] Berlim MT, Van Den Eynde F, Jeff DZ. Clinically meaningful efficacy and acceptability of low-frequency repetitive transcranial magnetic stimulation (rTMS) for treating primary major depression: a meta-analysis of Randomized, Double-blind and sham-controlled trials. Neuropsychopharmacology. 2013:38(4):543–551. 23249815 10.1038/npp.2012.237 PMC3572468

[ref7] Cardenas VA, Bhat JV, Horwege AM, Ehrlich TJ, Lavacot J, Mathalon DH, Glover GH, Roach BJ, Badran BW, Forman SD, et al. Anatomical and fMRI-network comparison of multiple DLPFC targeting strategies for repetitive transcranial magnetic stimulation treatment of depression. Brain Stimul. 2022:15(1):63–72. 34767967 10.1016/j.brs.2021.11.008 PMC8900427

[ref8] Cash RFH, Noda Y, Zomorrodi R, Radhu N, Farzan F, Rajji TK, Fitzgerald PB, Chen R, Daskalakis ZJ, Blumberger DM. Characterization of glutamatergic and GABAA-mediated neurotransmission in motor and dorsolateral prefrontal cortex using paired-pulse TMS–EEG. Neuropsychopharmacology. 2017:42(2):502–511. 27461082 10.1038/npp.2016.133 PMC5399228

[ref9] Castrén E . Is mood chemistry? Nat Rev Neurosci. 2005:6(3):241–246. 15738959 10.1038/nrn1629

[ref10] Castrén E . Neuronal network plasticity and recovery from depression. JAMA Psychiat. 2013:70(9):983. 10.1001/jamapsychiatry.2013.1 23842648

[ref11] Chen S, Liu X, Huang Z, Su F, Zhang W, Li J, Liu S, Ming D. Spatiotemporal connectivity maps abnormal communication pathways in major depressive disorder underlying gamma oscillations. Cereb Cortex. 2023:bhad204(15):9313–9324. 10.1093/cercor/bhad204 37310187

[ref12] Cooke SF, Bliss TVP. Plasticity in the human central nervous system. Brain. 2006:129(7):1659–1673. 16672292 10.1093/brain/awl082

[ref13] Delorme A, Makeig S. EEGLAB: an open source toolbox for analysis of single-trial EEG dynamics including independent component analysis. J Neurosci Methods. 2004:134(1):9–21. 15102499 10.1016/j.jneumeth.2003.10.009

[ref14] Delorme A, Palmer J, Onton J, Oostenveld R, Makeig S. Independent EEG sources are dipolar. PLoS One. 2012:7(2):e30135:1–14. 22355308 10.1371/journal.pone.0030135 PMC3280242

[ref15] Duman RS, Aghajanian GK. Synaptic dysfunction in depression: potential therapeutic targets. Science. 2012:338(6103):68–72. 23042884 10.1126/science.1222939 PMC4424898

[ref16] Duman RS, Aghajanian GK, Sanacora G, Krystal JH. Synaptic plasticity and depression: new insights from stress and rapid-acting antidepressants. Nat Med. 2016:22(3):238–249. 26937618 10.1038/nm.4050 PMC5405628

[ref17] Dunlop BW, Rajendra JK, Craighead WE, Kelley ME, McGrath CL, Choi KS, Kinkead B, Nemeroff CB, Mayberg HS. Functional connectivity of the subcallosal cingulate cortex and differential outcomes to treatment with cognitive-behavioral therapy or antidepressant medication for major depressive disorder. AJP. 2017:174(6):533–545. 10.1176/appi.ajp.2016.16050518 PMC5453828 28335622

[ref18] Ferreri F, Rossini PM. TMS and TMS-EEG techniques in the study of the excitability, connectivity, and plasticity of the human motor cortex. Rev Neurosci. 2013:24(4):431–442. 10.1515/revneuro-2013-0019 23907420

[ref19] Ferreri F, Pasqualetti P, Määttä S, Ponzo D, Ferrarelli F, Tononi G, Mervaala E, Miniussi C, Rossini PM. Human brain connectivity during single and paired pulse transcranial magnetic stimulation. NeuroImage. 2011:54(1):90–102. 20682352 10.1016/j.neuroimage.2010.07.056

[ref20] Fox MD, Buckner RL, White MP, Greicius MD, Pascual-Leone A. Efficacy of transcranial magnetic stimulation targets for depression is related to intrinsic functional connectivity with the subgenual cingulate. Biol Psychiatry. 2012:72(7):595–603. 22658708 10.1016/j.biopsych.2012.04.028 PMC4120275

[ref21] Frantseva MV, Fitzgerald PB, Chen R, Moller B, Daigle M, Daskalakis ZJ. Evidence for impaired long-term potentiation in schizophrenia and its relationship to motor skill leaning. Cereb Cortex. 2008:18(5):990–996. 17855721 10.1093/cercor/bhm151

[ref22] Gaynes BN, Lux LJ, Lloyd SW, Hansen RA, Gartlehner G, Keener P, Brode S, Evans TS, Jonas D, Crotty K, et al. Nonpharmacologic interventions for treatment-resistant depression in adults. Rockville (MD): Agency for Healthcare Research and Quality (US); 2011. 22091472

[ref23] Gray JP, Müller VI, Eickhoff SB, Fox PT. Multimodal abnormalities of brain structure and function in major depressive disorder: a meta-analysis of neuroimaging studies. AJP. 2020:177(5):422–434. 10.1176/appi.ajp.2019.19050560 PMC7294300 32098488

[ref24] Ilmoniemi RJ, Kičić D. Methodology for combined TMS and EEG. Brain Topogr. 2010:22(4):233–248. 20012350 10.1007/s10548-009-0123-4 PMC2800178

[ref25] Jaffe DH, Rive B, Denee TR. The humanistic and economic burden of treatment-resistant depression in Europe: a cross-sectional study. BMC Psychiat. 2019:19(1):247. 10.1186/s12888-019-2222-4 PMC6686569 31391065

[ref26] Jannati A, Oberman LM, Rotenberg A, Pascual-Leone A. Assessing the mechanisms of brain plasticity by transcranial magnetic stimulation. Neuropsychopharmacology. 2023:48(1):191–208. 36198876 10.1038/s41386-022-01453-8 PMC9700722

[ref27] Jung T-P, Makeig S, Humphries C, Lee T-W, McKeown MJ, Iragui V, Sejnowski TJ. Removing electroencephalographic artifacts by blind source separation. Psychophysiology. 2000:37(2):163–178. 10731767

[ref28] Kaneko N, Yokoyama H, Masugi Y, Watanabe K, Nakazawa K. Phase dependent modulation of cortical activity during action observation and motor imagery of walking: an EEG study. NeuroImage. 2021:225:117486. 33164857 10.1016/j.neuroimage.2020.117486

[ref29] Kedzior KK, Reitz SK, Azorina V, Loo C. Durability of the antidepressant effect of the high-frequency repetitive transcranial magnetic stimulation (rTMS) in the absence of maintenance treatment in major depression: a systematic review and meta-analysis of 16 double-blind, randomized, SHAM-CONTR: Review: depression and rTMS. Depress Anxiety. 2015:32(3):193–203. 25683231 10.1002/da.22339

[ref30] Kumar S, Zomorrodi R, Ghazala Z, Goodman MS, Blumberger DM, Cheam A, Fischer C, Daskalakis ZJ, Mulsant BH, Pollock BG, et al. Extent of dorsolateral prefrontal cortex plasticity and its association with working memory in patients with Alzheimer disease. JAMA Psychiat. 2017:74(12):1266–1274. 10.1001/jamapsychiatry.2017.3292 PMC6583382 29071355

[ref31] Lee P-S, Chen Y-S, Hsieh J-C, Su T-P, Chen L-F. Distinct neuronal oscillatory responses between patients with bipolar and unipolar disorders: a magnetoencephalographic study. J Affect Disord. 2010:123(1–3):270–275. 19755202 10.1016/j.jad.2009.08.020

[ref32] Levitt JG, Kalender G, O’Neill J, Diaz JP, Cook IA, Ginder N, Krantz D, Minzenberg MJ, Vince-Cruz N, Nguyen LD, et al. Dorsolateral prefrontal γ-aminobutyric acid in patients with treatment-resistant depression after transcranial magnetic stimulation measured with magnetic resonance spectroscopy. JPN. 2019:44(6):386–394. 31199104 10.1503/jpn.180230 PMC6821508

[ref33] Loheswaran G, Barr MS, Zomorrodi R, Rajji TK, Blumberger DM, Foll BL, Daskalakis ZJ. Impairment of neuroplasticity in the dorsolateral prefrontal cortex by alcohol. Sci Rep. 2017:7(1):5276. 28706262 10.1038/s41598-017-04764-9 PMC5509647

[ref34] Makeig S, Bell AJ, Jung T-P, Sejnowski TJ. Independent component analysis of electroencephalographic data. *Adv Neural Inf Process Syst*. 1996:8:145–151.

[ref35] McGrath CL, Kelley ME, Holtzheimer PE, Dunlop BW, Craighead WE, Franco AR, Craddock RC, Mayberg HS. Toward a neuroimaging treatment selection biomarker for major depressive disorder. JAMA Psychiat. 2013:70(8):821–829. 10.1001/jamapsychiatry.2013.143 PMC4413467 23760393

[ref36] Miller EK, Cohen JD. An integrative theory of prefrontal cortex function. Annu Rev Neurosci. 2001:24(1):167–202. 11283309 10.1146/annurev.neuro.24.1.167

[ref37] Noda Y, Cash RFH, Zomorrodi R, Dominguez LG, Farzan F, Rajji TK, Barr MS, Chen R, Daskalakis ZJ, Blumberger DM. A combined TMS-EEG study of short-latency afferent inhibition in the motor and dorsolateral prefrontal cortex. J Neurophysiol. 2016:116(3):938–948. 27226450 10.1152/jn.00260.2016 PMC5009207

[ref38] Noda Y, Zomorrodi R, Vila-Rodriguez F, Downar J, Farzan F, Cash RFH, Rajji TK, Daskalakis ZJ, Blumberger DM. Impaired neuroplasticity in the prefrontal cortex in depression indexed through paired associative stimulation. Depress Anxiety. 2018:35(5):448–456. 29637656 10.1002/da.22738

[ref39] Oostenveld R, Oostendorp TF. Validating the boundary element method for forward and inverse EEG computations in the presence of a hole in the skull. Hum Brain Mapp. 2002:17(3):179–192. 12391571 10.1002/hbm.10061 PMC6872070

[ref40] Pascual-Leone A, Freitas C, Oberman L, Horvath JC, Halko M, Eldaief M, Bashir S, Vernet M, Shafi M, Westover B, et al. Characterizing brain cortical plasticity and network dynamics across the age-span in health and disease with TMS-EEG and TMS-fMRI. Brain Topogr. 2011:24(3–4):302–315. 21842407 10.1007/s10548-011-0196-8 PMC3374641

[ref41] Pehrson A, Sanchez C. Altered γ-aminobutyric acid neurotransmission in major depressive disorder: a critical review of the supporting evidence and the influence of serotonergic antidepressants. DDDT. 2015:9:603–624. 10.2147/DDDT.S62912 PMC4307650 25653499

[ref42] Player MJ, Taylor JL, Weickert CS, Alonzo A, Sachdev P, Martin D, Mitchell PB, Loo CK. Neuroplasticity in depressed individuals compared with healthy controls. Neuropsychopharmacology. 2013:38(11):2101–2108. 23676792 10.1038/npp.2013.126 PMC3773676

[ref43] Premoli I, Castellanos N, Rivolta D, Belardinelli P, Bajo R, Zipser C, Espenhahn S, Heidegger T, Müller-Dahlhaus F, Ziemann U. TMS-EEG signatures of GABAergic neurotransmission in the human cortex. J Neurosci. 2014:34(16):5603–5612. 24741050 10.1523/JNEUROSCI.5089-13.2014 PMC6608220

[ref44] Price JL, Drevets WC. Neural circuits underlying the pathophysiology of mood disorders. Trends Cogn Sci. 2012:16(1):61–71. 22197477 10.1016/j.tics.2011.12.011

[ref45] Price RB, Shungu DC, Mao X, Nestadt P, Kelly C, Collins KA, Murrough JW, Charney DS, Mathew SJ. Amino acid neurotransmitters assessed by proton magnetic resonance spectroscopy: relationship to treatment resistance in major depressive disorder. Biol Psychiatry. 2009:65(9):792–800. 19058788 10.1016/j.biopsych.2008.10.025 PMC2934870

[ref46] Rajji TK, Sun Y, Zomorrodi-Moghaddam R, Farzan F, Blumberger DM, Mulsant BH, Fitzgerald PB, Daskalakis ZJ. PAS-induced potentiation of cortical-evoked activity in the dorsolateral prefrontal cortex. Neuropsychopharmacology. 2013:38(12):2545–2552. 23820586 10.1038/npp.2013.161 PMC3799076

[ref47] Rogasch NC, Sullivan C, Thomson RH, Rose NS, Bailey NW, Fitzgerald PB, Farzan F, Hernandez-Pavon JC. Analysing concurrent transcranial magnetic stimulation and electroencephalographic data: a review and introduction to the open-source TESA software. NeuroImage. 2017:147:934–951. 27771347 10.1016/j.neuroimage.2016.10.031

[ref48] Rossini PM, Burke D, Chen R, Cohen LG, Daskalakis Z, Di Iorio R, Di Lazzaro V, Ferreri F, Fitzgerald PB, George MS, et al. Non-invasive electrical and magnetic stimulation of the brain, spinal cord, roots and peripheral nerves: basic principles and procedures for routine clinical and research application. An updated report from an I.F.C.N. Committee. Clin Neurophysiol. 2015:126(6):1071–1107. 25797650 10.1016/j.clinph.2015.02.001 PMC6350257

[ref49] Rush AJ, Trivedi MH, Wisniewski SR, Nierenberg AA, Stewart JW, Warden D, Niederehe G, Thase ME, Lavori PW, Lebowitz BD, et al. Acute and longer-term outcomes in depressed outpatients requiring one or several treatment steps: a STAR*D report. Am J Psychiatry. 2006:163(11):1905–1917. 17074942 10.1176/ajp.2006.163.11.1905

[ref50] Sackeim H . The definition and meaning of treatment-resistant depression. J Clin Psychiatry. 2001:62:10–17. 11480879

[ref51] Shirazi SY, Huang HJ. More reliable EEG electrode digitizing methods can reduce source estimation uncertainty, but current methods already accurately identify Brodmann areas. Front Neurosci. 2019:13:1159. 31787866 10.3389/fnins.2019.01159 PMC6856631

[ref52] Somani A, Kar SK. Efficacy of repetitive transcranial magnetic stimulation in treatment-resistant depression: the evidence thus far. Gen Psych. 2019:32(4):e100074:1–8. 10.1136/gpsych-2019-100074 PMC6738665 31552384

[ref53] Spedding M, Neau I, Harsing L. Brain plasticity and pathology in psychiatric disease: sites of action for potential therapy. Curr Opin Pharmacol. 2003:3(1):33–40. 12550739 10.1016/s1471-4892(02)00008-5

[ref54] Stefan K . Induction of plasticity in the human motor cortex by paired associative stimulation. Brain. 2000:123(3):572–584. 10686179 10.1093/brain/123.3.572

[ref55] Stefan K, Kunesch E, Benecke R, Cohen LG, Classen J. Mechanisms of enhancement of human motor cortex excitability induced by interventional paired associative stimulation. J Physiol. 2002:543(2):699–708. 12205201 10.1113/jphysiol.2002.023317 PMC2290505

[ref56] Stefan K, Wycislo M, Classen J. Modulation of associative human motor cortical plasticity by attention. J Neurophysiol. 2004:92(1):66–72. 14724259 10.1152/jn.00383.2003

[ref57] Ter Braack EM, De Vos CC, Van Putten MJAM. Masking the auditory evoked potential in TMS–EEG: a comparison of various methods. Brain Topogr. 2015:28(3):520–528. 23996091 10.1007/s10548-013-0312-z

[ref58] Voineskos D, Blumberger DM, Zomorrodi R, Rogasch NC, Farzan F, Foussias G, Rajji TK, Daskalakis ZJ. Altered transcranial magnetic stimulation–electroencephalographic markers of inhibition and excitation in the dorsolateral prefrontal cortex in major depressive disorder. Biol Psychiatry. 2019:85(6):477–486. 30503506 10.1016/j.biopsych.2018.09.032

[ref59] Wada M, Nakajima S, Honda S, Takano M, Taniguchi K, Tsugawa S, Mimura Y, Hattori N, Koike S, Zomorrodi R, et al. Reduced signal propagation elicited by frontal transcranial magnetic stimulation is associated with oligodendrocyte abnormalities in treatment-resistant depression. jpn. 2022:47(5):E325–E335. 36104082 10.1503/jpn.220102 PMC9484613

[ref60] Williams JBW, Kobak KA. Development and reliability of a structured interview guide for the Montgomery-Åsberg depression rating scale (SIGMA). Br J Psychiatry. 2008:192(1):52–58. 18174510 10.1192/bjp.bp.106.032532

[ref61] Wolters A, Sandbrink F, Schlottmann A, Kunesch E, Stefan K, Cohen LG, Benecke R, Classen J. A temporally asymmetric Hebbian rule governing plasticity in the human motor cortex. J Neurophysiol. 2003:89(5):2339–2345. 12612033 10.1152/jn.00900.2002

[ref62] World Health Organization . Depression and other common mental disorders: global health estimates. Geneva: World Health Organization; 2017.

